# Transient Expression of Tetrameric Recombinant Human Butyrylcholinesterase in *Nicotiana benthamiana*

**DOI:** 10.3389/fpls.2016.00743

**Published:** 2016-06-16

**Authors:** Salem Alkanaimsh, Kalimuthu Karuppanan, Andrés Guerrero, Aye M. Tu, Bryce Hashimoto, Min Sook Hwang, My L. Phu, Lucas Arzola, Carlito B. Lebrilla, Abhaya M. Dandekar, Bryce W. Falk, Somen Nandi, Raymond L. Rodriguez, Karen A. McDonald

**Affiliations:** ^1^Department of Chemical Engineering, University of California, DavisDavis, CA, USA; ^2^Department of Chemistry, University of California, DavisDavis, CA, USA; ^3^Department of Plant Science, University of California, DavisDavis, CA, USA; ^4^Department of Plant Pathology, University of California, DavisDavis, CA, USA; ^5^Department of Molecular and Cellular Biology, University of California, DavisDavis, CA, USA; ^6^Department of Global HealthShare Initiative, University of California, DavisDavis, CA, USA

**Keywords:** butyrylcholinesterase, *Nicotiana benthamiana*, plant viral expression system, transient protein production, plant *N*-glycosylation, tetramerization

## Abstract

To optimize the expression, extraction and purification of plant-derived tetrameric recombinant human butyrylcholinesterase (prBChE), we describe the development and use of plant viral amplicon-based gene expression system; *Tobacco Mosaic Virus* (TMV) RNA-based overexpression vector (TRBO) to express enzymatically active FLAG-tagged plant made recombinant butyrylcholinesterase (rBChE) in *Nicotiana benthamiana* leaves using transient agroinfiltration. Two gene expression cassettes were designed to express the recombinant protein in either the ER or to the apoplastic compartment. Leaf homogenization was used to isolate ER-retained recombinant butyrylcholinesterase (prBChE-ER) while apoplast-targeted rBChE was isolated by either leaf homogenization (prBChE) or vacuum-extraction of apoplastic wash fluid (prBChE-AWF). rBChE from apoplast wash fluid had a higher specific activity but lower enzyme yield than leaf homogenate. To optimize the isolation and purification of total recombinant protein from leaf homogenates, an acidic extraction buffer was used. The acidic extraction buffer yielded >95% enzymatically active tetrameric rBChE as verified by Coomassie stained and native gel electrophoresis. Furthermore, when compared to human butyrylcholinesterase, the prBChE was found to be similar in terms of tetramerization and enzyme kinetics. The N-linked glycan profile of purified prBChE-ER was found to be mostly high mannose structures while the N-linked glycans on prBChE-AWF were primarily complex. The glycan profile of the prBChE leaf homogenates showed a mixture of high mannose, complex and paucimannose type N-glycans. These findings demonstrate the ability of plants to produce rBChE that is enzymatically active and whose oligomeric state is comparable to mammalian butyrylcholinesterase. The process of plant made rBChE tetramerization and strategies for improving its pharmacokinetics properties are also discussed.

## Introduction

Organophosphate (OP) nerve agents such as Sarin (Pohanka et al., 2013) have been used in recent history against civilian populations in Japan (Seto et al., 1999) and in Charbonneau and Nichols (2013). Their acute toxicity is due to an irreversible inhibition of human acetylcholinesterase (hAChE; Moshiri et al., 2012), which leads to accumulation of acetylcholine in the synaptic cleft followed by overstimulation of cholinergic receptors and death if left untreated (Trovaslet-Leroy et al., 2011). Although treatment options such as oximes, and atropine (Lenz et al., 2007) are available, they are usually administered as post-exposure prophylaxis and can have detrimental side effects such as damage to the central nervous system (Trovaslet-Leroy et al., 2011). A better treatment option is human butyrylcholinesterase, (hBChE), a bio-scavenger that specifically and irreversibly binds OP compounds with a MRT in the bloodstream of approximately 12 days (Duysen et al., 2002; Lenz et al., 2007; Saxena et al., 2008).

Human BChE is a glycosylated serine hydrolase that is made in the liver and circulates in the bloodstream catalyzing the hydrolysis of a variety of choline and non-choline esters (Darvesh et al., 2003). It binds to OP compounds irreversibly at a 1:1 stoichiometry (Çokuǧraş, 203). Circulating human BChE is a homotetrameric protein consisting of 85 kDa monomers comprised of 574 amino acids (Ngamelue et al., 2007). Monomers possess ten potential *N*-glycosylation sites of which, nine are known to be occupied (Kolarich et al., 2008). Tetramerization and sialylation of bi-antennary galactose residues on N-linked glycans of hBChE are known to be essential for long MRT in the bloodstream (Saxena et al., 2008). Because the concentration of hBChE in the blood is very low, extracting and purifying gram or kilogram quantities of hBChE from blood is impractical (Browne et al., 1998). This has led researchers to explore heterologous expression systems to produce functional recombinant hBChE in amounts that are affordable and practical for protecting civilian and military personnel from OP nerve agents. Some of these expression systems include transgenic goats, Chinese hamster ovary (CHO) cell culture, and human embryonic kidney cell culture (Nachon et al., 2002; Chilukuri et al., 2005; Huang et al., 2007). Although these systems are capable of producing functional rBChE, their drawbacks include high fermentation cost, slow cell growth rates, risk of viral infection of mammalian cell cultures and long lag time between gene transfer and lactation for transgenic animals (Houdebine, 2009; Thomas et al., 2011).

Plants, particularly *Nicotiana benthamiana* (*N. benthamiana)*, have been used to produce rBChE as well as other biopharmaceutical proteins (Goldstein and Thomas, 2004). Some of the advantages of the *N. benthamiana* expression system are its affordability, low risk of carrying human pathogens, its scalability and ability to glycosylate proteins (Raskin et al., 2002; Obembe et al., 2011; Xu et al., 2012). prBChE produced from transgenic *N. benthamiana* has been tested successfully as a bio-scavenger against multiple nerve agents (Geyer et al., 2010a,b). However, growth and scale up of transgenic lines can take several months (Garabagi et al., 2012) making it difficult to response rapidly to new chemical or biological challenges to human health. Alternatively, transient expression of prBChE in *N. benthamiana* can be achieved in 4–12 days, making transient expression systems well suited for rapid production of biodefense agents like hBChE (D’Aoust et al., 2010; Pogue et al., 2010; Schneider et al., 2014a,b). High level, rapid and transient expressing of target proteins can be achieved in *N. benthamiana* using a plant viral expression vector cloned in *Agrobacterium tumefaciens* (*A. tumefaciens*; Hefferon, 2012). Examples of plant viruses being used as viral expression vectors are *Tobacco Mosaic Virus*TMV (Lindbo, 2007; Kagale et al., 2012), *Cucumber Mosaic Virus* (CMV; Sudarshana et al., 2006; Hwang et al., 2012) and the Gemini virus-based vectors (Huang et al., 2010). Vacuum agroinfiltration is a fast and efficient mean for introducing recombinant *A*. *tumefaciens* harboring a gene of interest into plants. Transcription and translation of the gene starts within a few hours post-infiltration (Arzola et al., 2011).

**FIGURE 1:**
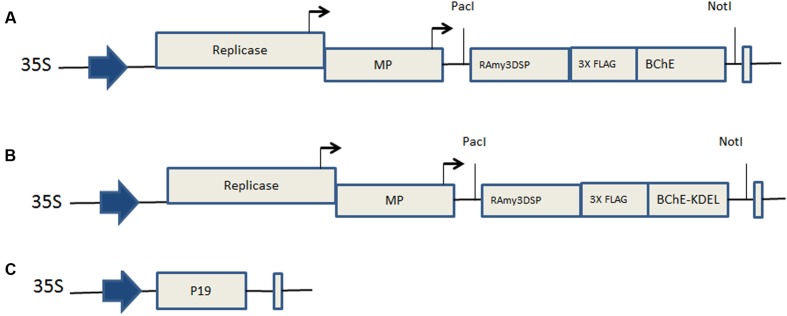
**Map of gene constructs. (A)** pTRBO-prBChE-KDEL, **(B)** pTRBO-prBChE, and **(C)** p35S-P19, 35S: Cauliflower Mosaic Virus (CaMV) promotor, *RAmy3DSP*: rice alpha-amylase 3D gene signal peptide, *Replicase*: replicase gene of tobacco mosaic virus (TMV), *MP*: movement protein, *BChE*: codon optimized sequences for the human butyrylcholinesterase gene. *3XFLAG*: specific amino acid codon sequences used for immunodetection and purification of the protein. *KDEL*: Codons encoding lysine, aspartic acid, glutamic acid, leucine, specifying the endoplamsmic retention sequence. P19: *P19* gene from *Tomato Bushy Stunt Virus* (TBSV).

The aim of this study was to use a plant viral expression system and purification strategies to express enzymatically active, tetrameric prBChE using transient agroinfiltration in *N. benthamiana* leaves. Two separate expression cassettes were designed to express rBChE in the ER (i.e., ER-retained; **Figure 1A**) or targeted to the apoplast (Apoplast-targeted; **Figure 1B**). Expression cassettes were cloned into the viral vector, TRBO, a TMV RNA-based overexpression vector (Lindbo, 2007). Expression vectors with their cassettes were separately cloned into *A. tumefaciens* and co-infiltrated into *N. benthamiana* leaves with the silencing suppressor P19. The levels, specific activities of differentially targeted prBChE (i.e., prBChE-ER vs. prBChE), and differentially extracted apoplast targeted prBChE (i.e., prBChE vs. prBChE-AWF), were estimated. Their N-linked glycan structures were determined. The results of this study indicate that prBChE, extracted from whole leaf homogenates was similar to the hBChE and eqBChE controls in terms of physiochemical properties, tetramerization and kinetic parameters.

## Materials and Methods

### Construction of Gene Expression Cassettes

Two gene expression cassettes were synthesized (GenScript, Piscataway Township, NJ, USA) for both intracellular and extracellular localization of prBChE. One cassette (*prBChE*+*KDEL*) was designed to retain prBChE in the ER (**Figure 1A**) while the other (*prBChE*) was used to target prBChE to the apoplastic space (**Figure 1B**). The *hBChE* sequence (Gene bank: NP_000046.1) lacking human signal peptide was codon optimized to a codon-adjusted index of 0.87 (from 0.76) to facilitate expression in *N. benthamiana.* In both expression cassettes (**Figures 1A,B**), the signal peptide of *hBChE* was replaced with the 75 base pair sequence coding for the rice alpha-amylase *RAmy3D* signal peptide (Gene bank: M59351). The *3xFLAG* tag sequence (Sigma–Aldrich, St. Louis, MO, USA) was inserted between the *RAmy3D* signal peptide and *hBChE* sequences in both cassettes.

### Construction of Viral Vector Expression Systems

To transcribe the two different rBChE expression cassettes, the TMV-based plant viral vector system, TRBO, was used. The binary vectors pTRBO-rBChE+KDEL (**Figure 1A**) and pTRBO-rBChE (**Figure 1B**) were made as follows. The *hBChE* cassette and the backbone vector, pTRBO, were digested with *AvrII* and *PacI* restriction enzymes after which the inserts were ligated to the pTRBO yielding pTRBO-rBChE+KEDL and pTRBO-rBChE viral expression vectors (**Figures 1A,B**). The recombinant vectors were transformed to DH5α *Escherichia coli* competent cells (Invitrogen, Carlsbad, CA, USA) following manufacturer specifications. *A. tumefaciens* EHA105 was used for transformation as described earlier (Shen and Forde, 1989). To prevent the RNAi-mediated gene silencing defense mechanism in plants, the silencing suppressor gene, *P19*, under the control of the 35S promoter (Arzola et al., 2011; **Figure 1C**) was co-infiltrated with the TRBO viral expression vector.

### Agroinfiltration and Incubation of *N. benthamiana* Plants

*Agrobacterium tumefaciens* (EHA105) cells containing pTRBO-rBChE+KDEL, pTRBO-rBChE, and p35S-p19, were grown in 5 ml LB media having the appropriate antibiotics for 24 h with shaking (250 rpm) at 28°C. The cultures were transferred into 200 ml LB media having 40 μM of acetosyringone (Sigma–Aldrich, St. Louis, MO, USA) grown overnight at 28°C with shaking (250 rpm). Cells were harvested by centrifugation at 2,600 *g* and resuspended in sterile, 10 mM MES buffer (Fisher Scientific, Santa Clara, CA, USA) pH 5.6, 10 mM MgCl_2_ and 150 μM acetosyringone to achieve an OD_600_ of 0.5. The TRBO expression cassette (pTRBO-rBChE+KDEL or pTRBO-rBChE) was mixed in a 1:1 volume ratio with the gene-silencing suppressor (p35S-p19). The mixed agrobacterium suspensions were incubated in the dark for 1 to 3 h before infiltration.

To validate and compare the viral expression of the two TRBO expression cassettes, three, 4–5 weeks old, *N. benthamiana* plants were used per experiment. The plants were grown in four-inch pots in a greenhouse under 16/8 h (light/dark) cycle. The potted plants were inverted and immersed in 600 ml of the agrobacteria solution containing 0.02% of Silwet-L-77 (Lehle seeds, Round Rock, TX, USA) and placed in a Nalgene container for vacuum application (-25 in Hg) for 2 min before releasing the vacuum. The agroinfiltrated plants were incubated in an environmental growth chamber at 90% humidity and 21°C for 6 days after which the leaves were cut at the petioles and harvested. The agroinfiltrated leaves were stored at -80°C for further analysis, or were immediately processed to recover apoplastic fluids.

### Extracting prBChE from agroinfiltrated *N. benthamiana* Leaves

Plant-derived BChE was extracted from *N. benthamiana* leaves using two extraction methods. First, leaf homogenates (prBChE-ER and prBChE) were extracted by grinding leaves frozen in liquid nitrogen with the extraction buffer TBS-1 (20 mM Tris-HCl, pH 8, 150 mM NaCl, 0.01% Tween 80) at a ratio of 1 g leaf tissue to 4 ml buffer. The extract was mixed for 30 min at 4°C before centrifugation at in 3,200 *g* for 30 min at 4°C. Supernatant was recovered and stored at 4°C for subsequent analysis.

### Apoplast Wash Fluid Recovery (AWF) of prBChE from Agroinfiltrated *N. benthamiana* Leaves

prBChE-AWF was extracted as follows: freshly harvested agroinfiltrated leaves were submerged in a harvest buffer, TBS-2 (20 mM Tris-HCl, pH 8, 150 mM NaCl, 0.02% Silwet L-77) and placed in a Nalgene container for a 2 min vacuum application. The infiltrated leaves were placed in 50 ml falcon tubes, and centrifuged for 15 min at 4°C at 250 g. The AWF was recovered and stored at 4°C.

### Quantification of Plant Soluble Proteins

Total soluble protein concentration was determined by the Bradford assay (Bio-Rad, Hercules, CA, USA) using bovine serum albumin (BSA; Sigma–Aldrich, St. Louis, MO, USA) as a standard. A standard curve was generated using BSA solutions ranging from 0.05 to 0.5 mg/ml. The absorbance of the standards and samples was measured at 595 nm with a SpectraMax 340C spectrophotometer (Molecular Devices, Sunnyvale, CA, USA).

### *In Vitro* Activity Assay of prBChE Protein

A modified Ellman assay (Ellman et al., 1961) was used to quantify the activity of butyrylcholinesterase. S-Butyrylthiocholine (BTCh) iodide (Sigma–Aldrich, St. Louis, MO, USA) and 5, 5′-dithiobis-2-nitrobenzoic acid (DTNB; Sigma–Aldrich, St. Louis, MO, USA) dissolved in 0.1 M phosphate buffer, pH 7.4 were used to make a working substrate solution at 0.5 and 0.267 mM, respectively. A volume of 150 μl of the working substrate solution was added to 50 μl of enzyme diluted with phosphate buffer in 96-well plates and the progress of the reaction was monitored in triplicates at 405 nm for 5 min at 25°C. A specific activity of 260 U/mg was used to convert from units of activity to mg rBChE.

### SDS-PAGE and Western Blot Analysis

Protein samples and controls were subjected to SDS-PAGE using a 4–15% gradient gel (Bio-Rad, Hercules, CA, USA) under non-reducing and reducing conditions with 5% β-mercaptoethanol (Bio-Rad, Hercules, CA, USA). Briefly, protein samples and controls were heated for 5 min at 95°C and electrophoresis performed for 35 min at 200 V. Gels were either stained in Coomassie Brilliant Blue G-250 (Bio-Rad, Hercules, CA, USA) or transferred to a 0.45 μm nitrocellulose membrane (Bio-Rad, Hercules, CA, USA) for 90 min at 100 V. Blots were blocked with 5% non-fat dry milk (NFDM) in (1X) PBS, pH 7.4. overnight and washed three times with (1X) PBST buffer at 5-min intervals. The blots were developed with either 1:2,500 dilution of monoclonal anti-FLAG M2-Peroxidase (HRP) antibody (Sigma–Aldrich, St. Louis, MO, USA) in 5% NFDM solution or with 1:200 mouse monoclonal anti-BChE antibody (D-5; Santa Cruz biotechnology, Santa Cruz, CA, USA) in 5% NFDM solution followed by 1:2000 goat anti-mouse HRP conjugated secondary antibody (Santa Cruz Biotechnology, Santa Cruz, CA, USA) in 5% NFDM solution. The blots were incubated for 1 h at room temperature in each antibody solution after which the blots were washed with (1X) PBST buffer three times for 5 min each. TMB stabilized substrate for horseradish peroxidase (Promega, Madison, WI, USA) was used as a color development substrate. Commercially available eqBChE (Sigma–Aldrich, St. Louis, MO, USA) and PEGylated goat-made butyrylcholinesterase (PEG-rBChE) kindly provided by Dr. Doug Cerasoli (USAMRICD) were used as controls.

### Purification of prBChE from Total Leaf Homogenates and AWF

To determine the optimal extraction buffer, the following two extraction buffers were used; 50 mM Tris-HCl, pH 8, 250 mM NaCl with 0.01% Tween 80 (TBS-3) and 50 mM citric buffer, pH 4, 250 mM NaCl with 0.01% Tween 80 (CBS). Frozen agroinfiltrated leaves with TRBO constructs with (*prBChE*+*KDEL*) and without KDEL sequence (*prBChE*) were used to purify prBChE-ER and prBChE from total leaf homogenate. Biomass was ground in liquid nitrogen at a ratio of 1 g:4 ml buffer and the amount of enzymatically active prBChE from total leaf homogenate and total soluble proteins were determined.

Based on the buffer screening experiments, CBS buffer was selected for protein recovery from homogenized leaves. The crude extracts (prBChE-ER and prBChE) were filtered through a 0.22 μm filter (EMD Millipore, Chicago, IL, USA) followed by filtration through a 30 kDa Minimate Tangential Flow Filtration Capsule (Pall Corporation, Ann Arbor, MI, USA). The concentrated retentate was loaded on ANTI-FLAG M2 affinity gel (Sigma–Aldrich, St. Louis, MO, USA) and the target protein was captured and eluted based on the manufacturer’s specifications. Similarly, the recombinant protein from the AWF was filtered, concentrated using a 30 kDa Amicon centrifugal filter units (EMD Millipore, Chicago, IL, USA) and purified using ANTI-FLAG M2 affinity gel (Sigma–Aldrich, St. Louis, MO, USA).

### Glycopeptide Analysis

#### Sample Preparation

Glycopeptide analysis of the different prBChE products was performed according to the method described by Nwosu et al. (2013) with a few modifications. Briefly, 5 μg of each prBChE sample was denatured for one h at 57°C in the presence of dithiothreitol. Alkylation was achieved by iodoacetamide addition and incubation in the dark. The alkylated samples were run on SDS-PAGE and Any-kD Mini Protean TGX gels (Bio-Rad, Hercules, CA, USA). After staining with Coomassie Brilliant Blue G-250 (Bio-Rad, Hercules, CA, USA) and rinsing in water, the targeted protein bands were excised from the gel, rinsed and destained using alternating washes (three cycles) of 100 mM ammonium bicarbonate (pH 8.0) and pure acetonitrile. Finally, the excised gel pieces were completely dried under vacuum and treated with 100 μl of a 0.005 μg/μl Pronase (Sigma–Aldrich, St. Louis, MO, USA) solution in 100 mM ammonium bicarbonate (pH 8.0). Digestion was allowed to proceed overnight at 37°C. The supernatant was withdrawn and dried in a SpeedVac (Genevac EZ-2, Stone Ridge, NY, USA) prior to the MS analysis.

#### Mass Spectrometry

Dried samples were reconstituted in 10 μl of deionized water and were analyzed using an Agilent 6520 Q-TOF mass spectrometer (Agilent Technologies, Santa Clara CA, USA). Tandem MS analysis of the samples was acquired in the positive mode in a data-dependent manner following LC separation on a microfluidic chip packed with porous graphite carbon (PGC), (Agilent Technologies, Santa Clara CA, USA). The two solutions pumped in these analyses consist of a binary solvent: A, 3.0% ACN/water (v/v) with 0.1% formic acid (FA); B, 90% ACN/water (v/v) with 0.1% FA. A flow rate of 3 μl/min of solvent A was used for sample loading with a 10 μl injection volume. Samples were eluted with 1% B (0.00–2.50 min); 1–16% B (2.50–20.00 min); 16–44% B (20.00–30.00 min); 44–99% B (30.00–35.00 min), and 99% B (35.00–45.00 min). Mass calibration was enabled using infused reference masses (ESI-TOF tuning mix G1969-85000, Agilent Technologies, Santa Clara CA, USA). For the tandem MS analysis, ions were subjected to collision-induced fragmentation using collision energies (*V*_collision_) that were dependent on the *m*/*z* value of the quasimolecular ions according to the equation *V*_collision_ = *m*/*z* (1.8/100 Da) V–2.4 V. The preferred charge states were set at 2, 3, and >3.

#### Data Analysis

Glycopeptides were assigned based on a combination of accurate mass measurement and tandem MS data. In-house developed software (Glycopeptide Finder; Strum et al., 2013) was used for rapid and automated assignment of the glycopeptides. The assignments were made within a specified tolerance level (≤20 ppm). To identify glycopeptides in the tandem MS data, product ion spectra were sorted by the presence of carbohydrate-specific oxonium fragment ions. The glycan moieties of the glycopeptides were confirmed by the presence of B-type and Y-type ions derived from the sequence of glycan fragmentations in the product ion spectra. However, the peptide moieties were mainly confirmed using accurate measurement of their masses in the tandem MS data. Identifications with a confidence level of 95% (based on the Glycopeptide Finder decoy analysis) were considered.

### Determination of Enzymatic Parameters of Plant Recombinant BChE Protein

The kinetic parameters of hBChE and prBChE were determined using a modified Ellman assay described previously. For these determinations, different concentrations of the substrate (BTCh) ranging from 0 to 7.5 mM were used in a 0.267 mM DTNB dissolved in 0.1 M phosphate buffer, pH 7.4. The progress of the reactions was monitored at the same conditions (*T* = 25°C and pH 7.4 for 5 min). Initial reaction rates were determined in triplicate at each substrate concentration and were plotted and a non-linear regression analysis was applied using GraphPad Prism ver.6 (La Jolla, CA, USA).

### Oligomeric State of prBChE Protein

The relative amounts of purified prBChE monomers, dimers, and tetramers were estimated by running hBChE and eqBChE (Sigma–Aldrich, St. Louis, MO, USA) as controls on a native gel. A 7.5% gel (Bio-Rad, Hercules, CA, USA) was used and electrophoresis was performed at 40 V for 7 h at 4°C. The gel was either stained with Coomassie Brilliant Blue G-250 (Bio-Rad, Hercules, CA, USA) or according to the method of Karnovsky and Roots (Karnovsky and Roots, 1964). The western blot was developed as described earlier.

## Results

### Quantifying Differentially Targeted prBChE in Agroinfiltrated *N. benthamiana* Leaves

**FIGURE 2:**
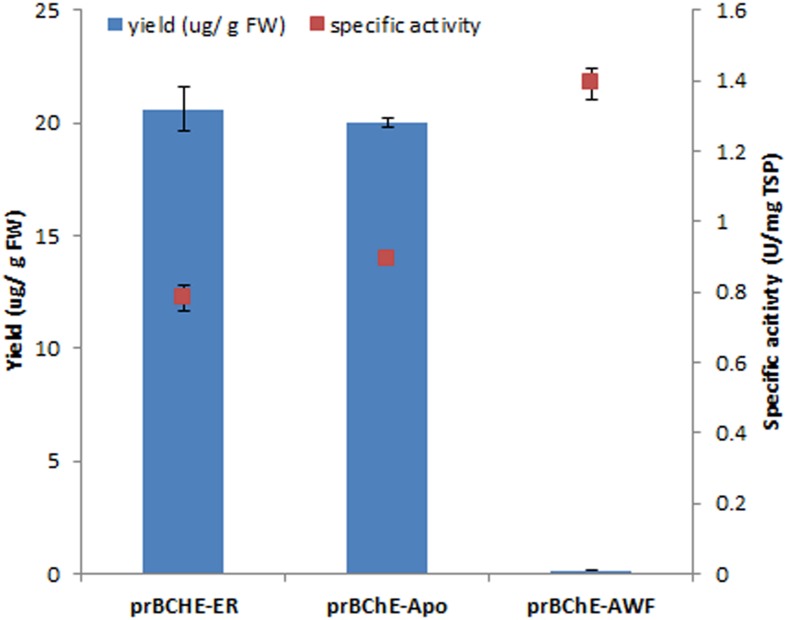
**Quantification of active prBChE protein in crude extracts using TRBO viral system as a function of sublocalization in plant cell and extraction methodology.** A modified Ellman assay was used to determine the accumulation level of active prBChE protein species localized in different sub-compartments in plant cells in *N. benthamiana* leaves agroinfiltrated with TRBO expression system. The apoplast-targeted prBChE was extracted either by leaf homogenization or collecting the AWF. Specific activity was determined as the ratio of active protein estimated from the Ellman assay relative to total soluble protein estimated by Bradford assay. All data plotted are the average of three independent measurements ± SD.

**FIGURE 3:**
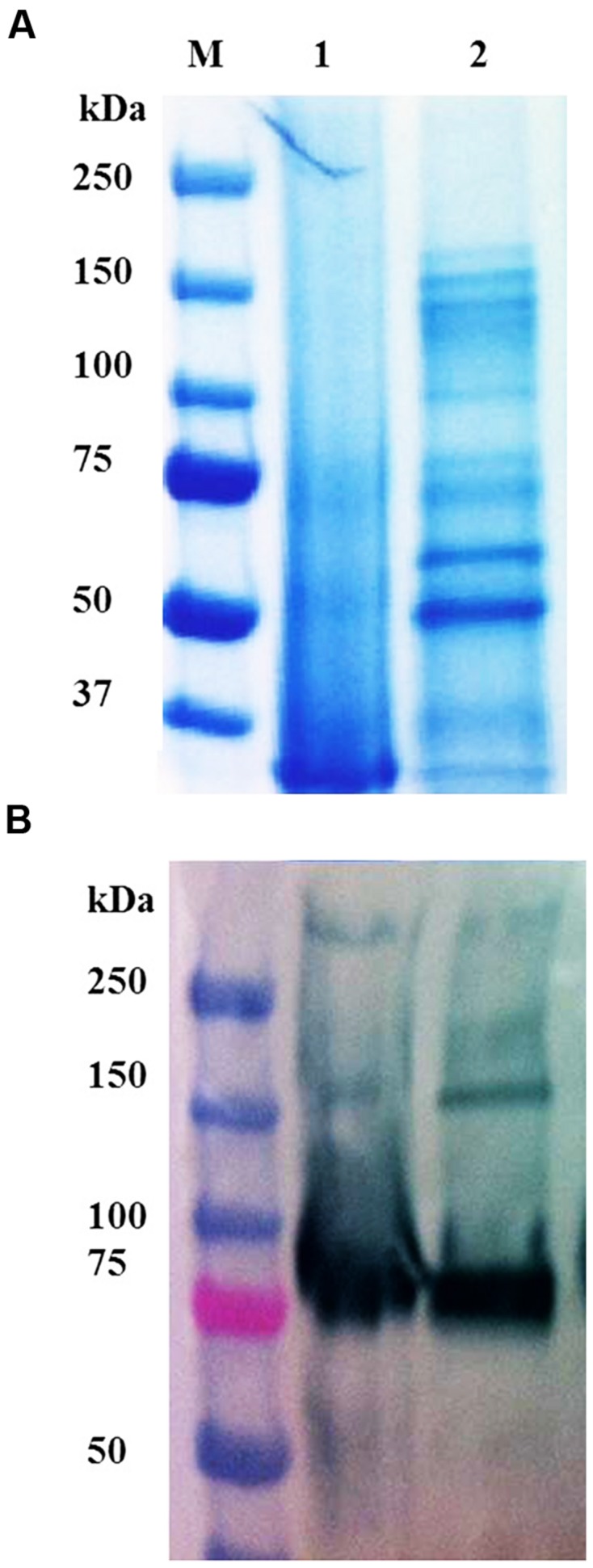
**(A)** SDS-PAGE and **(B)** Western blot of different variants of prBChE protein. **(A)** Coomassie stained gel with 7 μg total soluble protein (TSP) of prBChE-AWF (lanes 1) and total leaf extract of prBChE (lanes 2) loaded into each well under non-reduced conditions. Lane M shows the pre-stained protein molecular weight standards along with the molecular weight in kDa. **(B)** Western blot analysis using 1:2,500 anti-FLAG HRP conjugated antibody.

The amount of active and differentially targeted prBChE (i.e., with and without the KDEL sequence) produced by TRBO was determined. Total protein from leaf homogenates were extracted with TBS-1 and the level of active prBChE-ER was found to be approximately the same as the prBChE enzyme (**Figure 2**). In addition to isolating apoplast-targeted prBChE from agroinfiltrated *N. benthamiana* leaf homogenates, vacuum infiltration in TBS-2 was used to obtain prBChE in the apoplast wash fluid (prBChE-AWF). The amount of prBChE-AWF was two orders of magnitude lower than that recovered from whole leaf homogenates, which shows that this protein is poorly secreted to the apoplast (**Figure 2**). Due to the lower amount of total protein extracted from the AWF, the specific activity of the prBChE-AWF was approximately twice that obtained from leaf extracts (**Figure 2**). It is worth noting that and intercellular plant proteins like RuBisCo (large subunits around 50 kDa) were not detected in the AWF (**Figure 3A**). This may explain the higher specific activity obtained for prBChE-AWF compared to total leaf homogenates (**Figure 2**). **Figure 3** compares the prBChE obtained from the total leaf extract with that recovered from the AWF. As can be seen in **Figures 3A,B**, less prBChE was recovered using AWF method compared to prBChE obtained from homogenizing whole leaf tissue.

### Purification, SDS-PAGE Analysis and LC-MS/MS Analysis of Purified prBChE Protein

**FIGURE 4:**
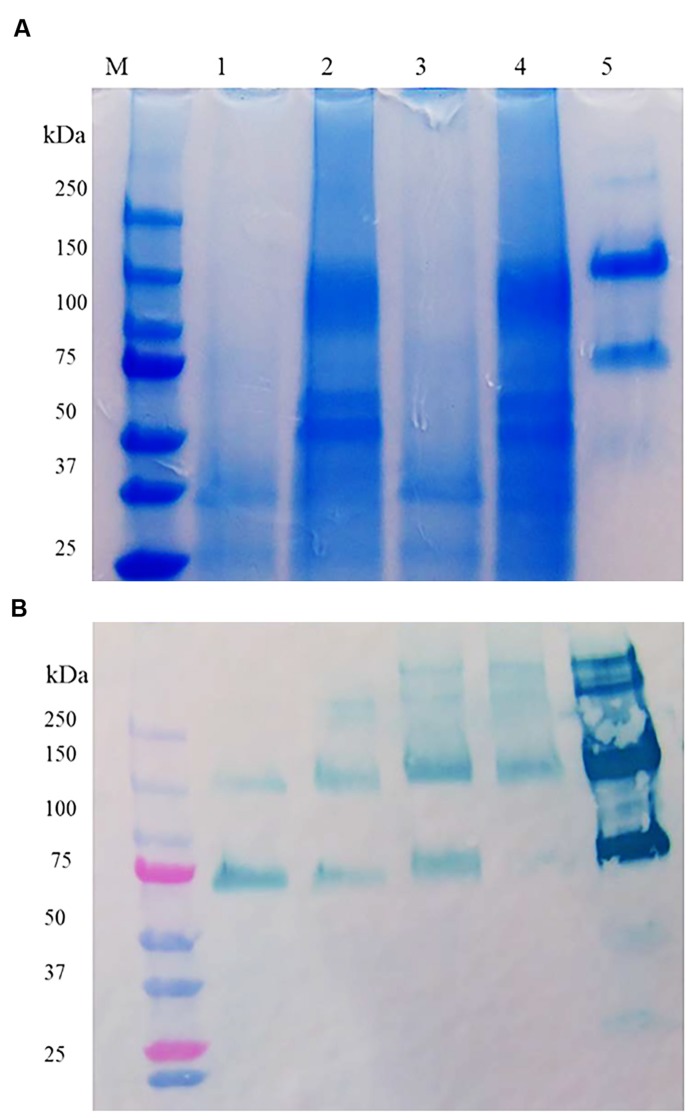
**(A)** SDS-PAGE and **(B)** Western blot of ER retained and apoplast targeted prBChE protein extracted using different extraction buffers. **(A)** Coomassie stained gel with 15 mU: lane1: (prBChE-ER pH 4 extract), lane 2: (prBChE-ER pH 8 extract), lane 3 (prBChE pH 4 extract), lane 4 (prBChE pH 8 extract), lane 5: (3 μg of equine BChE control) loaded under non-reduced conditions. Lane M shows the pre-stained protein molecular weight standards along with the molecular weight in kDa. **(B)** Western blot analysis using 1:200 mouse anti-BChE antibody and 1:2,000 goat anti-mouse HRP conjugated antibody.

Although the specific activity of prBChE-AWF was higher than that from leaf homogenates, it was not used for large-scale purification because of its low yield per unit mass. The two extraction buffers, TBS-3 (pH 8) and CBS (pH 4) were used to extract the leaf homogenates. As shown in **Figure 4A**, many intercellular proteins were extracted by total leaf homogenization using TBS-3 pH 8. Extracting prBChE in a high pH buffer and in the presence of many contaminating intercellular proteins (e.g., RuBisCo) lowered the specific activity of prBChE. A comparison of the extractability of enzymatically active prBChE using these two extraction methods is shown in **Figures 4A,B**. The SDS-PAGE analysis (**Figure 4A**) shows that RuBisCo was not extracted by the CBS buffer in contrast to the TBS-3 buffer extraction. As can be seen in **Figure 4A**, there were no prominent proteins migrating around the 50 kDa marker when extracting tobacco leaves with the acidic buffer.

**FIGURE 5:**
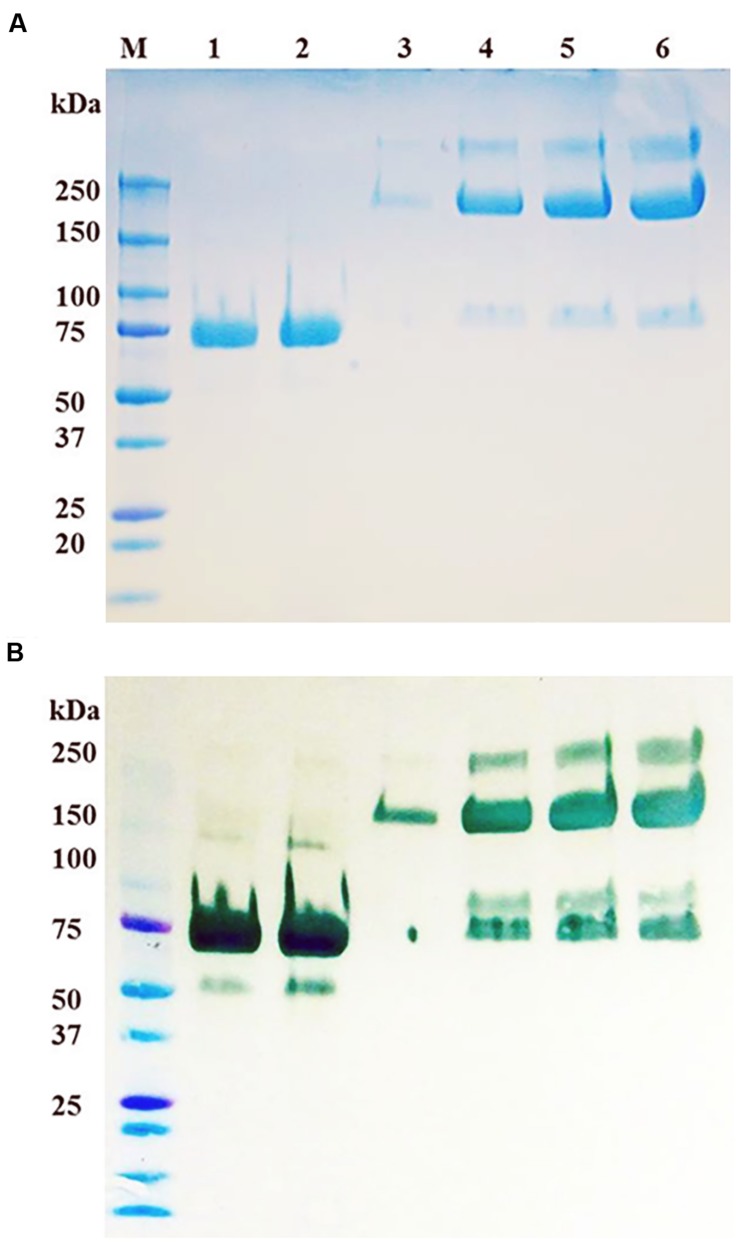
**(A)** SDS-PAGE and **(B)** Western blot of purified prBChE protein compared to serial dilutions of PEG-rBChE protein control. **(A)** Coomassie stained gel: lanes 1 and 2 contain duplicate loadings of 7 μg of prBChE protein. Lanes 3 to 6 contain serial dilutions of PEG-rBChE control (lane 3: 1, lane 4: 3, lane 5: 7, and lane 6: 10 μg), all loaded under reduced conditions. Lane M shows the pre-stained protein molecular weight standards along with the molecular weight in kDa. Because the goat recombinant BChE is PEGylated, its monomeric mobility on SDS-PAGE is approximately 200 kDa. However, the unPEGylated goat BChE co-migrates with prBChE as revealed by SDS-PAGE. **(B)** Western blot analysis using 1:200 mouse anti-BChE antibody and 1:2,000 goat anti-mouse HRP conjugated antibody.

Once the leaf homogenate extraction buffer was optimized, prBChE variants (i.e., prBChE-ER and prBChE) were extracted using the CBS buffer and purified using ANTI-FLAG affinity gel, in addition to purification of prBChE from the AWF. The purified prBChE protein variants were compared to a dilution series of PEG-rBChE protein standard. Although only 7 μg (based on activity) were loaded in lanes 1 and 2 (**Figures 5A,B**), the Western blot shows more intense bands for prBChE when compared to PEG-rBChE bands possibly indicating a less active form of the protein in the prBChE samples.

### Glycopeptide Analysis of Different Subcellular Localized prBChE Protein

**FIGURE 6:**
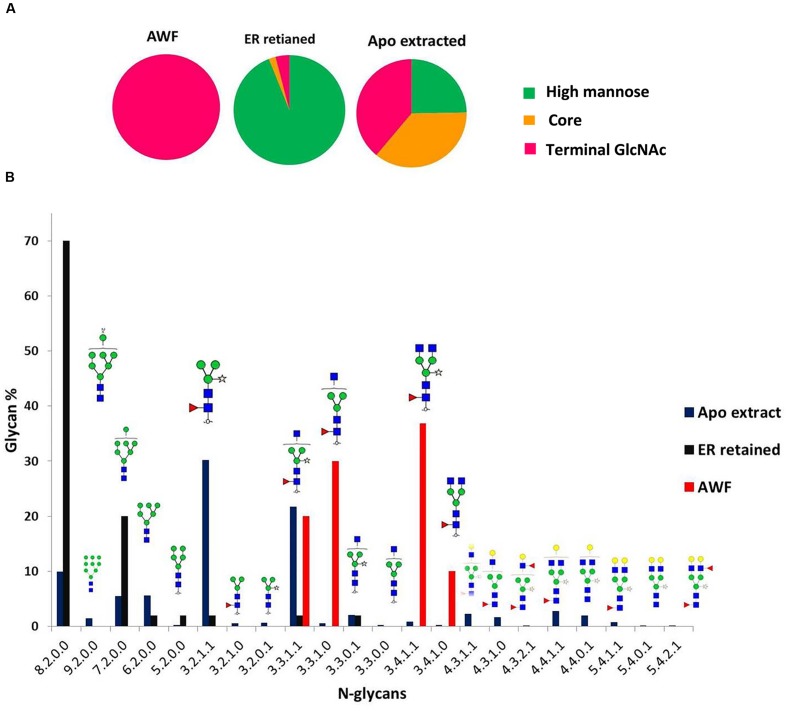
***N*-glycan distribution analysis of different prBChE species. (A)** Categories of *N*-glycans for each prBChE species are represented as pie charts. **(B)** Distribution of *N*-glycans of different prBChE species [prBChE-ER (ER), prBChE-AWF (AWF) and prBChE (Apo)] is shown on *y*-axis, while *x*-axis represents nomenclature of *N*-glycans as (Hex/HexNAc/Fucose/xylose).

For each sample, several glycopeptide compositions were identified. As some of the peptide moieties overlap in mass within the 20 ppm tolerance, site-specific information could not be determined. Nevertheless, as the glycan moieties were unambiguously identified, they were used to compare glycosylation patterns among the different prBChE samples. Extracted glycopeptide ion intensities were grouped and summed up by the composition of the glycan moiety and normalized for comparison. The variation in subcellular localization resulted in different glycosylation patterns on the recovered prBChE (**Figure 6**). As expected, the prBChE-ER showed mainly high-mannose structures (>90%) with small percentages of paucimannosidic-type N-linked glycan and complex N-linked glycans. The majority of the high mannose N-linked glycans had 6–8 mannose residues. Alternatively, prBChE-AWF consisted mostly of complex type N-linked glycans. Finally, prBChE yielded a mixture of different N-linked glycan types. Nearly 25% of the N-linked glycans were high-mannose structures, which are characteristic of the ER-retained prBChE protein. For prBChE, approximately 40% of the total observed N-linked glycans were complex N-linked glycans. A significant amount (approximately 35%) of paucimannosidic-type N-linked glycans was also found in the apoplast-targeted prBChE. The difference in the *N*-glycosylation between prBChE and prBChE-apoplast-targeted-AWF can be explained by differences in glycan processing as the protein moves along the secretory pathway from the ER to the trans-Golgi network.

### Enzymatic Properties and Oligomeric State of prBChE Protein

**FIGURE 7:**
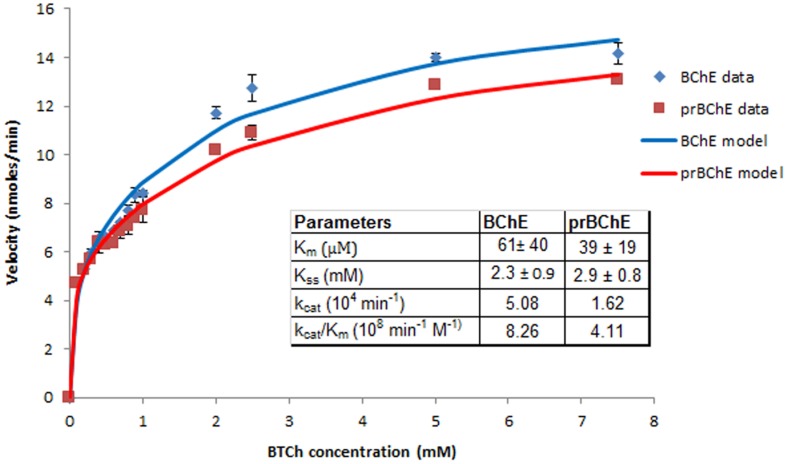
**Initial reaction velocity kinetics of BTCh hydrolysis by BChE enzyme.** The hydrolysis rate of BTCh substrate was monitored by measuring BChE activity (prBChE vs. human BChE control) over a large substrate concentration (10 μM–7.5 mM) in 0.1 M phosphate buffer pH 7.4, 0.267 mM DNTB.

The prBChE protein variant was selected for further characterization since it contains the highest amount of complex N-linked glycans, which are suitable for further *in vitro* modification including sialylation. The kinetics of butyrylthiocholine (BTCh) hydrolysis by prBChE was compared to the hBChE serum control. As previously described by Radic et al. (1993), BChE enzyme kinetics conforms to a substrate activation kinetic model when BTCh is used as a substrate. The rate of reaction was monitored over a large substrate concentration that ranged from 10 μM to 7.5 mM (**Figure 7**). Non-linear regression analysis shows that the *K*_m_ of prBChE for BTCh was (39 ± 19) μM, compared to the hBChE control (61 ± 40 μM). However, the turnover number of BTCh hydrolysis (*k*_cat_) for serum-derived hBChE was higher than that of prBChE.

**FIGURE 8:**
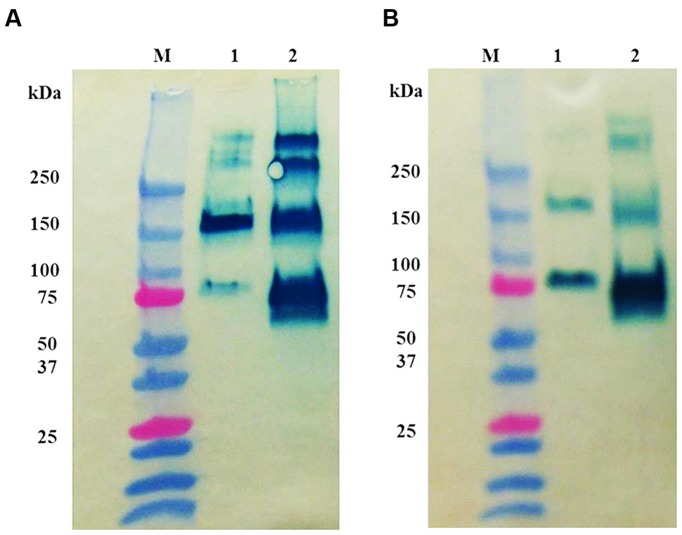
**Oligomer distribution analysis by Western blot using (A) non-reducing and (B) reducing conditions SDS-PAGE analysis.** Western blot analysis was developed using 1:200 mouse anti-BChE antibody and 1:2,000 goat anti-mouse HRP conjugated antibody of 0.25 μg of equine BuChE control (lane 1) and 0.25 μg pure prBChE protein (lane 2). Lane M shows the pre-stained protein molecular weight standards along with the molecular weight in kDa.

**FIGURE 9:**
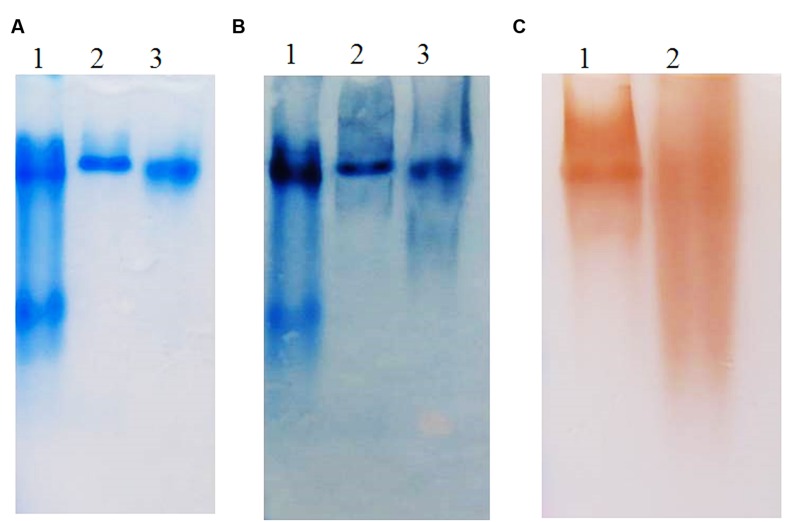
**(A)** Coomassie stained, **(B)** Western blot, and **(C)** activity stained Native gel analysis of prBChE, compared to BChE control. **(A,B)** Coomassie stained and Western blot 7.5% gel with 3 μg of purified prBChE (lane 3) compared to BChE mammalian serum enzymes (1.5 μg of human protein: lane 1, 3 μg equine protein: lane 2). **(C)** Activity stained of 0.25 mU pure prBChE (lane 2) and 0.25 mU equine control (lane 1).

**FIGURE 10:**
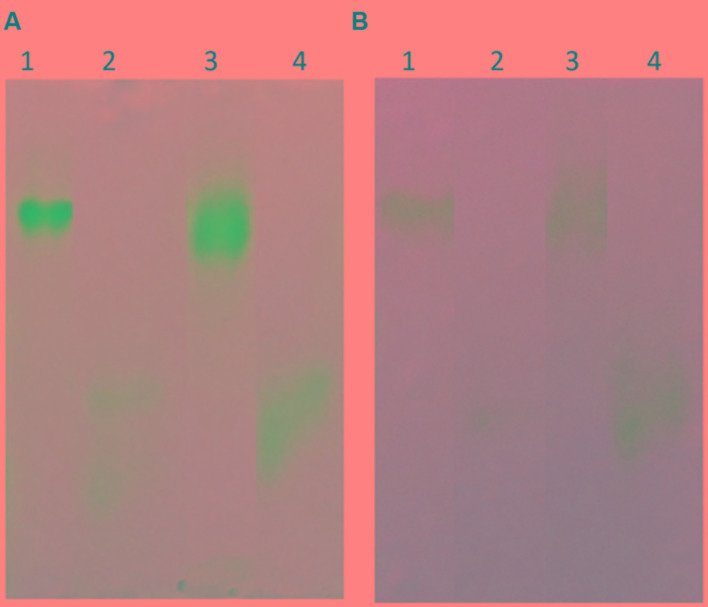
**(A)** Coomassie stained and **(B)** Western blot of Native gel analysis of denatured prBChE, compared to denatured equine BChE control. **(A)** Coomassie stained gel, lane 1: 1 μg equine BChE control under native conditions, lane 2: 1 μg of denatured equine BChE control under reducing conditions, lane 3: 1 μg prBChE under native conditions, lane 4: 1 μg of denatured prBChE under reducing conditions. **(B)** Western blot analysis developed with 0.5 μg equine BChE control and prBChE using 1:200 mouse anti-BChE antibody and 1:2,000 goat anti-mouse HRP conjugated antibody.

**FIGURE 11:**
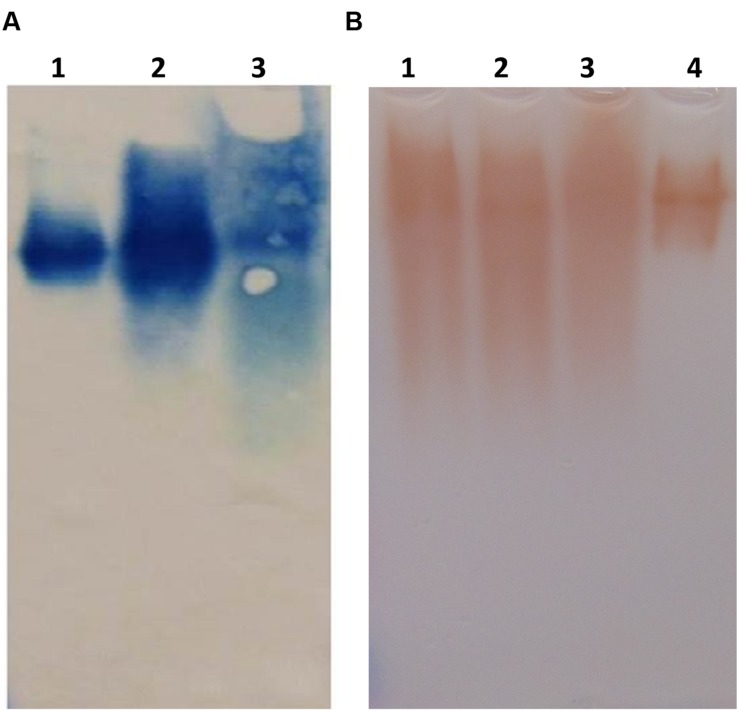
**(A)** Western blot analysis of a native gel of crude preparation of prBChE. Western blot analysis was performed using 1:200 mouse anti-BChE antibody and 1:2,000 goat anti-mouse HRP conjugated antibody of crude extract preparation of 0.25 μg prBChE (lane 3) compared to 0.25 μg of equine control (lane 1) and 0.25 μg pure prBChE protein (lane2). **(B)** Native gel analysis of different prBChE variants. 45 mU of prBChE-ER (lane 1), prBChE (lane 2), prBChE-AWF (lane 3) and equine control (lane 4) were loaded to a 7.5% gel and stained for BChE activity according to the method of Karnovsky and Roots (1964).

Different oligomers of prBChE were observed by fractionating the protein by SDS-PAGE under reduced and non-reduced conditions and on corresponding immunoblots (**Figure 8**). Under non-reduced conditions, the prBChE was observed migrating as monomeric, dimeric, and possible tetrameric structures. While under reducing conditions, the monomers of prBChE were the predominant form due to the reduction of intermolecular disulphide bonds. To estimate the various types and proportions of prBChE oligomers, native PAGE analysis was performed under non-denaturing conditions. The recombinant protein showed an oligomeric state similar to that of the human and equine serum controls. Almost all the protein was in the tetrameric state (**Figures 9A–C**). Denatured prBChE and the equine serum control were analyzed using native PAGE and the migration patterns were found to be similar (**Figure 10**). This indicates that the native form of prBChE is a tetrameric protein. Furthermore, native gel electrophoresis was used to separate the various molecular species in the crude leaf homogenate. This revealed only tetrameric prBCHE relative to the eqBChE (**Figures 11A,B**).

## Discussion

In the quest for a safe, abundant and affordable source of rBChE from plants, studies by the Lockridge group (Blong et al., 1997; Ngamelue et al., 2007; Biberoglu et al., 2012; Larson et al., 2014) and others (Masson et al., 2003; Schneider et al., 2014a,b) have focused attention on the desirable pharmacokinetic (PK) properties of prBChE. For prBChE, desirable PK properties include functionality, reduced risk of immunogenicity-allergenicity and longer MRT in the bloodstream afforded by sialylation of terminal galactose residues on N-linked glycans (Schneider et al., 2014a) and tetramerization of the BuChE monomers. The production of large quantities of any plant-made biopharmaceutical lacking its key PK properties does little to provide effective therapeutic solutions to natural or man-made threats to human health.

In this report, we described the expression of rBChE in *N. benthamiana* in a manner that yielded enzymatically active protein that was greater than >95% tetrameric (**Figures 9–11**). Enzymatic activity and electrophoretic mobility, on native gels, was similar to that seen with eqBChE and hBChE controls (**Figures 9–11**). In terms of yield, the TRBO expression vector produced prBChE-ER at 0.78 U/mg total soluble protein, which was comparable to that obtained by Geyer et al. (2010a; geometric mean = 0.7 U/mg protein) for the expression of ER retained prBChE from transgenic *N. benthamiana*.

Our findings are generally consistent with those of (Geyer et al., 2010b) who showed that approximately 50% of their prBChE purified from *N. benthamiana* leaf homogenates was enzymatically active tetramer. Like (Schneider et al., 2014b), the presence of a FLAG tag on the N-terminus of our prBChE did not affect its enzymatic activity or ability to be purified. Although we observed only tetrameric prBChE on native gels (**Figure 11B**) in all of our extracts (ER, AWF, and Apo), we cannot compare our findings to those of (Schneider et al., 2014b) because of significant different between our protocols. For example, the protocols of (Schneider et al., 2014b) used non-reducing gel electrophoresis to estimate oligomerization status while we used native gels. In addition, different viral-based expression vectors were used. At present, there is no clear explanation for these differences in oligomeric status and yield between our findings and (Schneider et al., 2014b). Additional experiments are underway to identify the factor(s) responsible for these differences.

A more fundamental question is how monomers of an 85 kDa mammalian enzyme oligomerize into tetramers in the milieu of the plant ER. Although determining the mechanism of prBChE tetramerization *in planta* is beyond the scope of this report, two possible explanations should be considered. First, oligomerization of mammalian proteins like hBChE may tetramerize in the ER or during transport through the secretory pathway. This can be supported by *N*-glycan profiling of the purified prBChE, which shows 25% of its *N*-glycans with high mannose structures (**Figure 6**). Alternatively, prBChE dimers may form tetramers as cellular contents are released by homogenization into the extraction environment. Regardless of which possibility is correct, both possibilities require that the entropic penalty of organizing prBChE into highly enthalpic tetramers, be paid by reducing ordered water molecules in and around prBChE as it proceeds along the assembly pathway (Ali and Imperiali, 2005; Fatmi and Chang, 2010). Furthermore, whether in plant or mammalian cells, the oligomerization of proteins is highly complex, sensitive to numerous intracellular and extracellular factors such as; protein concentration, temperature, pH, hydrogen and ionic bonding, hydrophobic interactions, phosphorylation, domain swapping and ligand binding (Ali and Imperiali, 2005; Gotte and Libonati, 2014). While BChE dimers are formed from intermolecular disulfide bonds at Cys571, tetramer assembly involves non-covalent affects and interactions such as those described above. *A priori*, this makes tetramerization of BChE highly sensitive to these factors and difficult to predict and control. Interestingly, the pH of the ER and trans-Golgi network has been found to be a gradient from the ER to the plasma membrane of 7.2 to 5.2 in mammals (Paroutis et al., 2004) and 6.8 to 5.2 in plants (Bassil et al., 2012). Whether a pH gradient contributes in any way to BChE tetramerization is a matter of speculation at this time.

Regarding ligand binding, it has been well documented that hBChE contains a C-terminal tetramerization domain that interacts non-covalently with small, naturally occurring PRP 12 to 21 residues long *in vivo* the to promote tetramerization (Nicolet et al., 2003; Dvir et al., 2004; Pan et al., 2009; Biberoglu et al., 2012). It was further shown that these PRPs do not merely catalyze tetramerization but become part of the BChE tetrameric complex. In another study, synthetic PRPs ranging from 15 to 50 residues were used to promote *in vitro* tetramerization of CHO cell-derived rBChE in a concentration-, temperature-, and time-dependent manner (Larson et al., 2014). These results are consistent with studies on the oligomerization of multi-subunit enzymes that showed that ligand binding lowers the conformation transition barrier and helps the protein conformation shift from its inactive to its active form (Ali and Imperiali, 2005; Fatmi and Chang, 2010). This interaction may help explain the role of PRPs in the tetramerization of rBChE from mammalian sources.

The implications of these studies are important for those planning to use plants to express prBChE in that it suggests the tetramerization of hBChE is dependent on its non-covalent interaction with a PRP, the likes of which have not been observed in plants. Although hydroxyproline-rich, O-linked, glycopeptides (HRGP) are found in both monocotyledonous and dicotyledonous plants, they are largely interspersed repeats of hydroxyproline (Albenne et al., 2009), not the tandem repeats found in the PRPs associated with native human BuChE. Another interesting feature of plant HRGPs is that the hydroxyproline repeats are frequently interspersed with charged amino acids (e.g., lysine and glutamic acid) making them sensitive to changes in pH. The fact that tetrameric prBChE can be detected in *N. benthamiana* leaf extracts (Geyer et al., 2010a,b; Schneider et al., 2014b) raises the possibility that plant HRGPs can substitute for mammalian PRPs to promote tetramerization of prBuChE dimers. We hypothesize that the plant ER can pay the entropic penalty of oligomerization by drawing upon its vast array of processes, mechanisms, signals and possibly HRGPs, to tetramerize prBChE in a concentration-, temperature-, and pH-dependent manner. Studies are currently underway to investigate these independent variables on oligomerization of BChE in plants tissues and plant cell suspension cultures.

## Conclusion

The objective of this study was to investigate methods for expressing active, tetrameric prBuChE in *N. benthamiana* leaf homogenates and AWF with the goal of increasing its desirable PK properties. We were able to produce prBChE variants using TRBO viral expression vector and purified to near homogeneity using affinity chromatography. The pure prBChE was found to be a tetramer protein as shown by Coomassie stained SDS-PAGE gels and native gel electrophoresis. We understand that there are other strategies for making prBChE a “bio-better” therapeutic using glycan engineering of plant N-linked glycans. For example, the galactose residues on the terminal N-linked glycans on prBChE can serves as substrate for *in vitro* (Malekan et al., 2013), or *in vivo* (Paccalet et al., 2007; Schneider et al., 2014a) sialylation to help extend the MRT of prBChE in human blood. Also, *N. benthamiana* fucosyl-transferase and xylosyl-transferase (ΔFT/XT) knock-down lines can be used to produce prBChE with reduce immunogenicity and/or allergenicity (Schneider et al., 2014b). While the *3XFLAG* was used to facilitate purification of prBChE, it can be removed enzymatically prior to its use as an injectable therapeutic to reduce risk of immunogenicity. Lastly, the expression of biopharmaceuticals like prBChE in plant cell suspension cultures using well-established fermentation technology may be the shortest path to achieving scalability, affordability and regulatory approval of a plant-made biopharmaceutical like prBChE. We believe that all expression, purification and production options should be explored to help meet the global demand for the next generation of safe, efficacious and affordable biopharmaceuticals derived from plants.

## Author Contributions

Conceived and designed the experiments: KM, SA, KK, SN, RR, CL, AD, and BWF. Preformed the experiments: SA, BH, AG, MSH, AMT, MLP, and LA. Analyzed the data: SA, KM, KK, SN, RR, AG, and CL. Wrote the paper: SA, KM, RR, and AG. All authors read, revised, and approved the MS.

## Conflict of Interest Statement

KM is a cofounder of Inserogen, Inc., a plant-based biotechnology startup company with a focus on the development of orphan biologics for replacement therapy. The other authors declare that the research was conducted in the absence of any commercial or financial relationships that could be construed as a potential conflict of interest.

## Abbreviations

Apo: apoplast
AWF: apoplast wash fluid
BChE: native human butyrylcholinesterase
CBS: citric buffered saline
ER: endoplasmic reticulum
eqBChE: equine butyrylcholinesterase
HRGP: hydroxyproline-rich glycopeptides
MRT: mean residence time
PK: pharmacokinetics
prBChE: plant-made recombinant butyrylcholinesterase
PRP: proline-rich peptides
rBChE: recombinant butyrylcholinesterase
TBS: tris buffered saline
